# Cognitive Restoration in Children Following Exposure to Nature: Evidence From the Attention Network Task and Mobile Eye Tracking

**DOI:** 10.3389/fpsyg.2019.00042

**Published:** 2019-02-05

**Authors:** Matt P. Stevenson, Richard Dewhurst, Theresa Schilhab, Peter Bentsen

**Affiliations:** ^1^Center for Outdoor Recreation and Education, University of Copenhagen, Copenhagen, Denmark; ^2^Interacting Minds Center, Aarhus University, Aarhus, Denmark; ^3^Department of Education, Aarhus University, Aarhus, Denmark; ^4^Health Promotion Research, Steno Diabetes Center Copenhagen, Gentofte, Denmark

**Keywords:** Attention Restoration Theory, executive attention, nature walk, intra-individual variance, effort allocation, state regulation

## Abstract

Exposure to nature improves cognitive performance through a process of cognitive restoration. However, few studies have explored the effect in children, and no studies have explored how eye movements “in the wild” with mobile eye tracking technology contribute to the restoration process. Our results demonstrated that just a 30-min walk in a natural environment was sufficient to produce a faster and more stable pattern of responding on the Attention Network Task, compared with an urban environment. Exposure to the natural environment did not improve executive (directed) attention performance. This pattern of results supports suggestions that children and adults experience unique cognitive benefits from nature. Further, we provide the first evidence of a link between cognitive restoration and the allocation of eye gaze. Participants wearing a mobile eye-tracker exhibited higher fixation rates while walking in the natural environment compared to the urban environment. The data go some way in uncovering the mechanisms sub-serving the restoration effect in children and elaborate how nature may counteract the effects of mental fatigue.

## Introduction

A rapidly expanding evidence base has shown exposure to natural environments (NEs) supports cognitive functioning in adults (e.g., Berman et al., 2008); however, very few studies have explored the effect in children (Stevenson et al., 2018). This is concerning given the potential role nature could play in maintaining optimal cognitive functioning during modern childhoods that are increasingly technological, competitive, and stressful (Song, 2014; Birenbaum et al., 2015).

The framework for understanding the links between NEs and cognitive function is known as Attention Restoration Theory (ART; Kaplan, 1995). According to its advocates, the mental fatigue one experiences throughout the day can be overcome by spending time in NEs. One’s capacity to direct attention toward goal-related information, while ignoring irrelevant information, is limited and diminishes with use over time. In contrast to built environments (BEs), NEs contain stimuli that capture attention in an involuntary manner with irrelevant stimuli being ignored more readily and the capacity-limited attentional system being depleted more slowly (Kaplan, 1995). This environmentally induced switch to bottom-up attentional processing is believed to lead to the restoration of abilities that require top-down directed attention. Nature is argued to contain “softly” or intrinsically fascinating stimuli which can be appreciated in a contemplative way without mental effort. That is, thoughts and perceptions are not actively pulled by benign natural stimuli in a way that would otherwise interrupt trains of thought. It has recently been suggested that the reduction in effort required to wilfully control bottom-up attention may foster a beneficial form of internal reflection, or mind-wandering, that supports creative thinking patterns through a simultaneous reduction in the effort required to navigate attention between thoughts and impressions (Williams et al., 2018). Finally, ART also predicts that optimally restorative environments should evoke a sense of *being away* from fatiguing situations; are *extensive* and rich enough to be engaging; and are *compatible* with one’s desires (Kaplan, 1995).

The premise that natural stimuli induce a characteristic processing pattern has been explored using lab-based eye-tracking equipment and digital photographs (Berto et al., 2008; Valtchanov and Ellard, 2015). Both studies reported that natural scenes were viewed with fewer fixations than urban scenes, which was interpreted as visual attention being captured in a less-effortful manner. Although the explanations of these differences in visual processing rely heavily on the cognitive mechanisms outlined in ART, to date, there have been no studies linking real-world visual processing of natural environments and subsequent changes in cognitive performance—everything has previously been assessed with photographs in the lab, which likely overlooks the kind of immersive state which ART argues nature brings about. Therefore, the importance of differential sensory processing and sense of engagement, and its association with cognitive performance, remains theoretical.

A recent meta-analysis found that exposure to NEs can lead to improvements in tasks that tap working memory, cognitive flexibility, and attentional control (Stevenson et al., 2018), which are considered the three core executive functions that give rise to higher cognitive operations, such as planning and problem solving (Diamond, 2013). It has been predicted that gains in executive functioning after exposure to a NE occur due to the restoration of directed attention, a common resource tapped during performance of the tasks used to assess these abilities (Kaplan and Berman, 2010). The constructs of directed attention and executive attention both describe the ability to focus attention while simultaneously ignoring distraction and are considered to be synonymous (Diamond, 2013). In adults, this ability is selectively improved after exposure to NEs when assessed using a task that separates executive attention from other processes, namely, the Attention Network Task (ANT) (e.g., Berman et al., 2008). The ANT produces a performance indicator (executive score) that most closely captures the directed attention construct described in ART (Ohly et al., 2016); indicators of non-executive attention performance (orienting and alerting scores); and general or temporal indicators of performance, such as speed (mean reaction time) and stability (standard error of reaction time). Therefore, utilizing the ANT in studies exploring restorative environments may extend current understandings of the restoration effect, as the mechanisms behind the effect, particularly in children, appear to be obscured.

Two large epidemiological studies involving the ANT suggested children between the ages of four and 10 years may benefit from NEs in ways distinct from adults. Dadvand et al. (2015, 2017) found that the amount of greenspace around the homes and schools of children was related to a reduction in intra-individual variability in reaction time (IIVrt), indexed using standard error of reaction time. Dadvand et al. (2017) study also found an association between greenspace and a reduction in IIVrt and omission errors on Conner’s Kiddies Continuous Performance Test. Further investigations in the same population revealed significant increases in gray matter volume in bilateral prefrontal areas, and increases in white matter volume in right prefrontal areas related to exposure to greenspace (Dadvand et al., 2018). This is a particularly important finding for ART given that stimulus-based (i.e., bottom-up) and voluntary (i.e., top-down) attention networks converge in lateral prefrontal areas (Asplund et al., 2010), and white matter maturation is associated with reduced IIVrt (Tamnes et al., 2012). These studies suggest that, for children, cognitive resources regulating temporal aspects of performance, such as state regulation (Wiersema et al., 2005), may be more sensitive to NEs than a more specific executive resource. This is supported by the only two randomized controlled trials to investigate the effect in typically developing children. Jenkin et al. (2017) found children were worse at delaying gratification of a future reward after exposure to digital BEs compared to natural environments, while selective attention, measured by the Stroop Task, remained unchanged. Schutte et al. (2017) found that children responded faster in the Continuous Performance Task and Go/NoGo Task after walking in a natural environment but did not show improvements in spatial working memory and response inhibition. While it appears children and adults may receive separable restorative benefits from exposure to natural environments, the exact mechanisms driving the effect remain obscured without additional measures that go beyond behavioral performance, like mobile eye tracking.

### The Present Study

The first aim of the present study was to investigate whether exposure to a NE improves children’s performance on the ANT, owing to a real-time or immediate restoration mechanism. We used a randomized crossover design to test whether children show directed attention improvements related to restoration effects. Additionally, we were interested in whether the previously reported long-term effects on IIVrt can be replicated and attributed to a restorative process, using a standard ART protocol, where mental fatigue is induced prior to environmental exposure. The final aim was to investigate whether visual processing differed between the two environments, as indexed by eye movement recordings using mobile eye tracking technology in the real world (Tobii Pro II eye-tracking classes).

We predicted that children’s performance on the ANT would be improved after a short walk in a natural environment. However, contrary to past studies with adult participants, we predicted improvement to be indexed by temporal performance indicators (reaction time, standard error of reaction time), rather than attention-specific performance indicators (executive score) We suggest such a pattern of results would reflect the restoration of a broader, regulatory, or energetic resource, rather than the restoration of directed attention, which has been attributed to performance gains in adults (Berman et al., 2008). Further, we predicted children would view the natural environment with fewer and longer fixations than the BE, reflecting the engagement of an attentional system that requires less top-down control. Finally, we predicted a significant association between cognitive performance and fixation measures, suggesting that gains in cognitive performance are to some extent dependant on the visual processing of NEs.

## Materials and Methods

### Study Design

We conducted a semi-randomized crossover trial using environment type as a within-subjects treatment variable with two levels (i.e., natural and built). For the first 23 participants (69.7%), the order of environment was randomized; however, this was not possible for the remaining participants due to restraints of their school schedules.

### Participants

**FIGURE 1:**
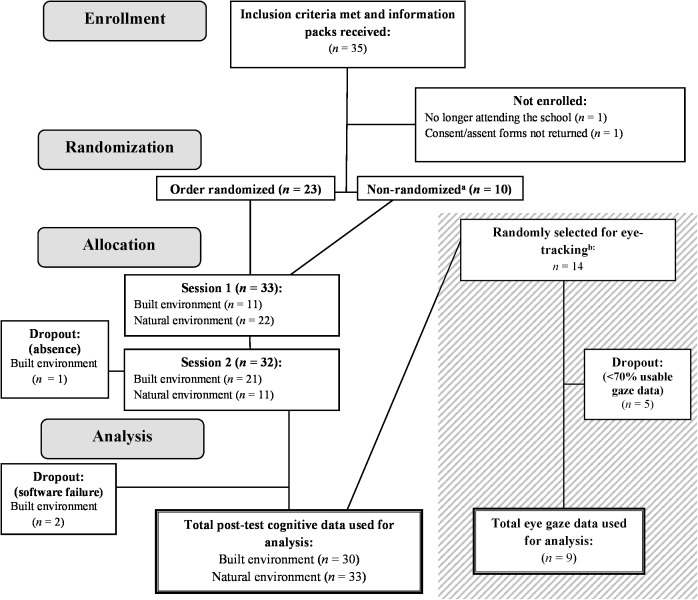
CONSORT flow diagram. A CONSORT-inspired flowchart depicting participant numbers through phases of the study from recruitment to data analysis, including participant dropout points. The details of the eye-tracking data collection are presented in the gray box. ^a^Computerized randomization of environment order was only possible for 23 out of 33 participants (69.7%) due to logistic restraints caused by the participants’ academic schedule. ^b^Randomization of environment order for eye-tracking data collection was not possible for the same reason. All eye-tracking data were collected first in the natural environment, followed by the built environment (BE).

Thirty-three children (*m* = 12.03 years; 60.6% female) were recruited from an independent school in Naestved, Denmark, from which a teacher had responded to a call for participants for a similar study. Figure 1 illustrates the flow of participants from recruitment to data analysis. Participants were recruited across three grade levels with the age of participants ranging from 10 to 14 years. The study was introduced to the school during a parent assembly. The information presented was carefully worded to not disclose hypotheses or any details that may influence behavior during the study. The research was presented as a study of walking, while details that would reveal the expectations from the environment and our hypotheses were withheld. Discussions and information letters included phrases such as “walking in two different environments” to avoid using language that could be construed as nature-positive. Interested parents were given an information pack that included written consent and assent forms. Only participants whose parents had signed consent forms after discussing participation took part in the study. In line with national and institutional standards, there was no formal ethical approval required for this type of study.

Most participants were somewhat familiar with both environments used in the study. Participants with a current diagnosis of any behavioral or cognitive disorder were excluded. There were no further criteria for exclusion. Cognitive data from two participants could not be used due to computer failures, and one participant did not complete the entire protocol. Fourteen participants (*m* = 12.43 years; 71.4% female) were randomly selected from the original sample to take part in the data collection for eye-tracking measures.

### Measures and Equipment

#### The Attention Network Task (ANT)

Participants performed a child-friendly version of the ANT which measures three distinct attention systems (Rueda et al., 2004). The task contained three blocks of 48 trials, preceded by a 12-trial practice block with feedback on performance. Each block lasts around 5 min with a rest period offered between blocks. During each trial, participants responded to the direction a centrally presented cartoon fish was facing. For trials that measure alerting ability, a cue is presented to inform the participants a target will appear. Performance on these trials is contrasted with trials that do not present a cue. The alerting cue facilitates performance, which is reflected in shorter reaction times during cued trials. For the trials that measure orienting ability, a spatial cue is presented to show the place in which the target stimulus will appear. Performance on these trials is contrasted with trials where a neutral cue is presented. The spatial cue facilitates performance, resulting in shorter reaction times. The trials that measure executive attention ability are similar to a common Flanker Task (Mullane et al., 2009). During these trials, the target fish is flanked by four other fish pointing in congruent or incongruent directions. Trials with incongruent flanker stimuli are contrasted with trials with congruent flanker stimuli. Incongruent flankers are associated with slower reaction times, reflecting the need to overcome a higher level of distraction. Four main outcome variables derived from behavioral ANT data. The executive attention score (EXE) was included as an index of directed attention ability; standard error of reaction time on all correct trials (SERT); mean reaction time on all correct trials (mRT); and total accuracy (ACC) were included as temporal or general performance indicators.

#### The Perceived Restorativeness Scale for Children (PRS-C II)

The PRS-C II is an age-adjusted version of the Perceived Restoration Scale that was created to obtain subjective ratings of restorative environmental components (Bagot et al., 2007), as described in ART (Kaplan, 1995). The scale contains 15 likert-style items that produce an overall perceived restorativeness score derived from sub-scales including fascination, compatibility, extent, being away – psychologically (e.g., novelty), and being away – physically (e.g., escape). The original scale was developed with reference to environments specific to children, such as school (Bagot, 2004); however, we adjusted the wording in order to relate the questions to the two environments used in our study. We replaced the original words “school ground” (Bagot et al., 2007) with “forest” (*Danish:* “*skov*”) and “streets” (*Danish:* “*gader*”) to capture features of the two environments. For example, “*The things I like to do can be done in the forest*” and “*The things I like to do can be done in the streets*.” The two versions of the PRS-C II were translated to Danish and were found to have similar levels of reliability (Cronbach’s alpha: “forest” version, α = 0.87; “streets” version, α = 0.81) for total perceived restorativeness as other translated versions (e.g., α = 0.84, Collado and Corraliza, 2017). Each participant completed one PRS-C II questionnaire for each environment. The total perceived restorativeness scores were used during analysis.

#### Tobii Pro 2 Mobile Eye-Tracking Glasses

Eye-tracking data was collected at a sampling rate of 50 Hz using Tobii Pro 2 glasses and processed using Tobii Pro Lab software. The glasses record first-person video through a wide-angle lens centered between the eyes (the scene camera). The coordinate system for eye-gaze localization relates the pupil-center corneal-reflection-vector to positions from the scene camera, using an infra-red light source (Holmqvist et al., 2011). Eye movement types were categorized using the Velocity-Threshold Identification Fixation Filter. This filter uses velocity threshold algorithm where raw gaze data under a certain velocity level are deemed to be fixations, while gaze data over a certain velocity level are deemed to be saccades. A threshold of 30 degrees per second was used for classifying fixations.

### Environmental Exposures

**FIGURE 2:**
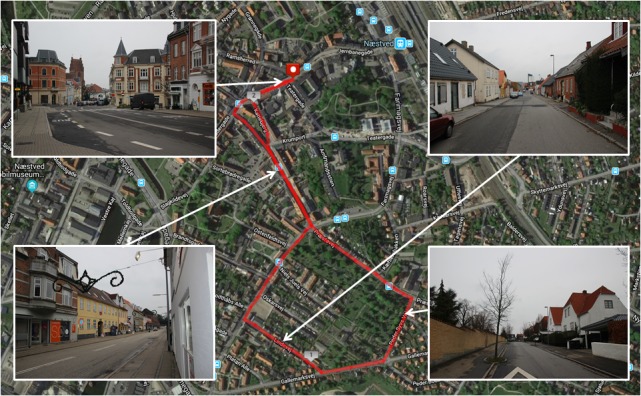
Route map and photographs of the built environment exposure walk. Aerial map of Næstved, Denmark, where the route for the built environment exposure walk was selected (marked in red). A youth centre was the location of the cognitive testing area and the start/end point for the route. The route map was created using MapMyRun (http://www.mapmyrun.com).

Figure 2, 3 present the two environments used, which were located within the local municipality, and familiar to most participants. For cognitive data collection, participants walked for 30 min on looped routes that were matched as closely as possible for pedestrian traffic, length, and gradient. Despite best attempts to match urban and natural walking environments, the urban walk contained a short section (i.e., 1–2 min) where noise was generated by a construction site and contained notably more traffic in the first and last 3–4 min. Natural features were present on the urban route, including planted trees and gardens, although these were sparsely distributed. Similarly, man-made features were sparsely distributed on the natural route, including fencing, sheds, vehicles, houses, benches, and signage. The natural walking environment was a rural area called Myrup. The route consisted of rolling grass fields, walking tracks through young pine trees and rocks, farmland, and forest containing mostly beech and birch. The route formed a complete loop with no backtracking. The built walking environment was a quiet neighborhood area adjacent to the main town area of Naestved. The route initially passed shops to lead out of the town area and then circumnavigated a cemetery with high stone walls, passing a mostly residential area with a primary school. The loop contained around 8 min of backtracking. The walking routes for eye-tracking data collection consisted of following the original routes for 5 min, before backtracking for 5 min to the beginning.

**FIGURE 3:**
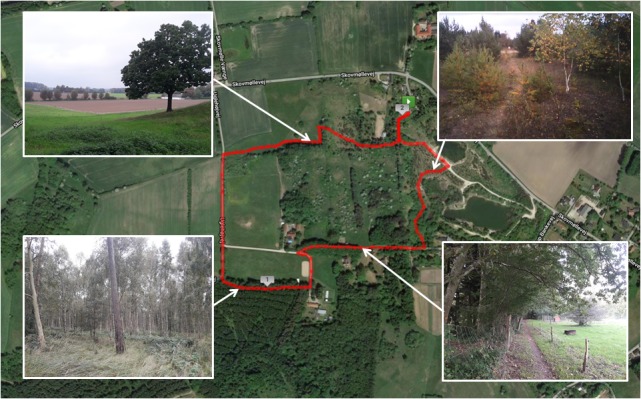
Route map and photographs of the natural environment exposure walk. Aerial map of Myrup, Denmark, where the route for the natural environment exposure walk was selected (marked in red). A rural property located in Myrup was the location of the cognitive testing area and the start/end point for the natural environment route. The route map was created using MapMyRun ().

### Procedure

Data was collected over two sessions, conducted on Mondays and Thursdays of two consecutive weeks. Participants were randomly assigned to a day, containing one of two permutations of environmental exposure. Those assigned to the Monday sessions received the exposure order *natural, built;* those assigned to the Thursday sessions received the exposure order *built, natural.* Time of testing was controlled by ensuring each participant was tested at roughly the same time (∼08:30; ∼10:30; or ∼12:30) during each session, and sessions were canceled due to any amount of rain.

In groups of four-five, participants sat at their own work station in a quite testing area, where the walks began and ended. They first performed the Digit Span Forward and Digit Span Backward Tasks, which lasted 12–15 min. This task was chosen to induce cognitive fatigue as it has been shown to be among the most sensitive measures to the restoration effect and therefore likely depletes relevant cognitive resources. To ensure participants allocated sufficient effort during these tasks there was a competition and prize for best performance, which participants were informed of prior to completion. Participants then performed the ANT (Fan et al., 2002) to obtain baseline measures of cognitive performance in their fatigued state. The ANT took around 15 min. Immediately after, participants walked in groups of four or five for 30 min along a predefined route in either a natural or BE. During the walk, there were 2 min periods where participants were encouraged to not communicate, to relax, and experience their surroundings. These periods were broken by periods where the experimenter asked the participants to comment on what they have seen, heard, smelt, and felt whilst walking. This activity was included to help the participants feel comfortable and ensure there was a degree of mindful interaction between the participants and their environment. In this way, the ratio between talking and silent periods was not strictly standardized and was applied at the discretion of the experimenter based on the atmosphere and engagement of the group. There were between six and eight talking periods during the walks, depending on the number of impressions offered by the group. Upon returning from the walk, participants sat at their workstations and performed the ANT once more to give post-treatment measures of cognitive performance. Finally, participants completed the version of the Perceived Restorativeness Scale for Children that corresponded to the environment in which they had walked.

Eye-tracking data were collected during two additional sessions in which the order could not be randomized. Therefore, all participants walked in the natural environment first for the eye-tracking data collection. After calibration procedures, 14 randomly selected participants walked one at a time with the experimenter wearing Tobii Pro II glasses following a 10-min segment of the routes used during cognitive data collection. Participants were instructed to try to relax and experience the environment as they normally would. All participants walked the same segment of the original routes with only a researcher to accompany them. Data collected for the first 2 min of the eye-tracking walk were discarded to allow for habituation of wearing the glasses. Data epochs were segmented from 2-min into the recording until a particular feature on the route was present. This resulted in slight variation in recording lengths due to variation in the speed at which participants walked. Therefore, fixation rate per minute was used as an outcome measure to normalize for this. We also took average length of fixation as an additional eye tracking measure.

### Statistical Analysis

The effect of environment type on cognitive performance was analyzed using an “intention-to-treat” approach. Initially, the data was visually inspected to remove outlier responses that were under 200 ms (<0.003%). Pre- and post-test averages from the four measures of cognitive performance (EXE, ACC, mRT, SERT) were then calculated for all participants across all three blocks of the ANT.

Mobile eye-tracking in outdoor environments is associated with challenges that lead to reduced pupil identification and therefore lost signal. For example, regular natural lighting causes constriction of the pupil, and high levels can cause squinting (Evans et al., 2012). To ensure the most reliable eye-tracking data were analyzed, we excluded participants with gaze sample rates lower than 70%—that is, for all remaining participants no more than 30% of raw sample data was lost for the entire recording period.

Paired samples *t*-tests were conducted to compare perceived restorativeness scores, total number of fixations, and average duration of fixations across the two environments. A series of linear mixed models (LMM) were used to analyse how post-test cognitive performance varied as a function of environment, while controlling for pre-test (baseline) scores. LMMs were used to account for the dependence of data points and the occurrence of missing data, which was done through restricted maximum likelihood estimations. The initial models fitted to the cognitive data included environment (i.e., natural, built) as a fixed factor, subject as a random factor, and pre-test (baseline) scores as a covariate. Follow-up analyses were conducted in a stepwise manner to reduce residual variance from other variables collected during the study. These included age, gender, order of environment; restoration tendency (PRS-C nature minus PRS-C built), number of fixations per minute, and average fixation length. Effect sizes (Cohen’s *d*) were calculated for significant main effects.

### Power Analysis

Power studies require knowledge about the expected effect size as well as of the variation of the outcome variable. These figures were difficult to obtain as there were no previous studies with children reporting the effect of short-term exposure to natural environments on ANT performance at the time of this study’s conception. Until Dadvand et al. (2015) the environmental effect was assumed to be found using the executive attention score. Therefore, we conducted a tentative power analysis based on adult literature (e.g., Berman et al., 2008) where we estimated a decrease of 20 ms in RT in executive score for the natural environment and no decrease for the BE. Estimating variance is more difficult as it varies greatly between studies and populations. Nevertheless, a standard deviation of around 20 was selected based on available evidence of children’s performance on the ANT (e.g., Adólfsdóttir et al., 2008). With these values, 40 children are required to get a power of 90% (when the test is carried out with significance level 5%).

## Results

### Participant Characteristics and Eye-Tracking

Thirty-two participants from the initial 33 recruited completed the experimental protocols in both environments, while one participant did not attend an assigned session due to sickness. Participants with ANT accuracy scores that were more than two and a half standard deviations below the mean were deemed to have unreliable task performance and their data was excluded. Based on this criterion, post-test data from a single session were excluded for two participants. One pre-test score from another participant was removed due to computer failure. These exclusions resulted in total of 33 and 30 participants included in the analyses for natural and BEs, respectively. The differences in age and gender between the two conditions were negligible (see Table 1). As predicted, the participants perceived the natural environment as being more restorative than the BE, *t*(29) = 2.56, *p* = 0.016.

**Table 1:** Participant details and eye-tracking data.

Participant characteristics	Natural environment	Built Environment	*p*-value	ES
**ANT data**				
Sample size^∗^	33	30		
Age	12.03 (1.21)	12.00 (1.23)		
Gender (male/female)	13/20	12/18		
Perceived restorativeness Scale	44.80 (8.69)	39.57 (10.84)	**0.016**	0.180
**Eye-tracking data**				
Sample size^†^	9	9		
Minutes analyzed	5.72 (0.74)	5.78 (0.86)	0.855	
Fixations per minute	157.97 (11.47)	145.01 (10.70)	**0.033**	1.168
Fixation duration	181.78 (26.22)	184.44 (32.93)	0.807	


*ES: effect size (Cohen’s d). ^∗^Data from the built environment sessions of three participants were excluded due to non-completion (n = 1) and computer software failure (n = 2). Paired samples t-test on Perceived Restorativeness Scale data conducted with a total sample size of n = 30. ^†^Data collected from a randomly selected sub-sample [original subsample n = 14; five participants excluded from analysis due to unreliable eye-tracking data (gaze sample rates under 70%)]. Bold type indicates a significant effect (p < 0.05).*

From the 14 participants randomly selected for eye-tracking data collection, five were excluded due to poor quality data (gaze sample rate under 70%). Checks were performed to ensure the group used for eye-tracking data analysis was representative of the initial participants recruited. The participants whose data was included in the analysis did not differ from the other participants in terms of age (*p* = 0.094), gender (*p* = 0.283), or PRS scores for the natural (*p* = 0.163) or BEs (*p* = 0.982). Moreover, the total number of minutes used for eye-tracking analysis did not differ between the two environments (*p* = 0.855). Participants made a greater number of fixations per minute while walking in the natural environment than in the BE, *t*(8) = 2.567, *p* = 0.033; however, there was no difference in the average duration of fixations between the two environments, *t*(8) = -0.253, *p* = 0.801.

### The Effect of Environment on Cognitive Performance

**FIGURE 4:**
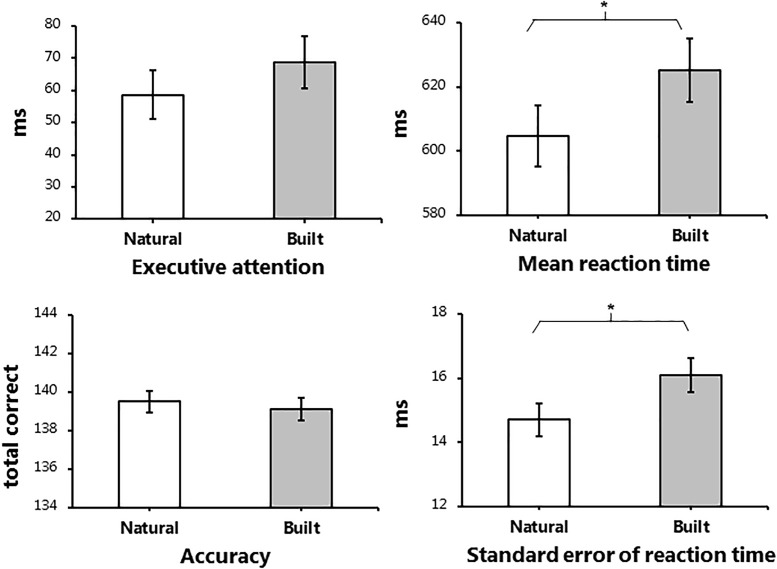
Adjusted post-walk data for performance of the Attention Network Task. Adjusted means and standard errors of the initial base model applied during linear mixed model analysis for the four outcome measures of the Attention Network Task. The post-test data displayed in the figure were adjusted using baseline data as a covariate. Subject was included in the base model as a random factor. ms: milliseconds. Significant main effects of environment are marked, ^∗^*p* < 0.05.

The main effect of environment was of primary interest during the analysis. Figure 4 displays the post-walk means and standard deviations for the four outcome measures of interest, adjusted for baseline performance. The initial base model used for analysis included baseline score as covariate and subject as a random factor. As shown in Tables2, 3, participants did not show improvements in executive attention or accuracy when exposed to the natural environment. However, exposure to the natural environment was associated with significantly faster reaction times on correct responses, β = -20.387, *p* = 0.024, and improved stability of performance, β = -1.387, *p* = 0.013. For follow-up analyses, we included additional factors in order to reduce variance in the base model. In addition to baseline performance, we explored the influence of age, gender, order of environment, and restorativeness tendency score, plus fixations per minute and average fixation length, in a step-wise manner. While the effect of baseline performance was significant in all base models, the additional factors applied in follow-up analyses did not significantly influence cognitive performance, across all measures.

**Table 2:** Means and standard errors for Attention Network Task (ANT) outcome measures (adjusted for baseline performance).

ANT outcome measures	Natural environment	Built environment	Main effect *P*	(Envi) ES
Executive attention score (EXE)	58.69 (7.46)	68.73 (7.96)	0.361	
Accuracy (ACC)	139.50 (0.56)	139.12 (0.60)	0.647	
Mean reaction time (mRT)	604.73 (9.45)	625.12 (9.83)	**0.024**	-0.383
Standard error of reaction time (SERT)	14.70 (0.50)	16.08 (0.53)	**0.013**	-0.486


*ES = effect size (Cohen’s d). Bold type indicates a significant effect (p < 0.05).*

**Table 3:** Linear mixed models exploring the effect of environment on Attention Network Performance in typically developing children.

	Model 1	
	β (95% CI)	*p*-value
**EXE**		
Envi	-10.034 (-31.856, 11.788)	0.361
Baseline	0.424 (0.126, 0.722)	**0.006**
Intercept	42.437 (17.839, 67.035)	**0.001**
AIC	632.984	
AIC Model 0	640.447	
**ACC**		
Envi	0.380 (-1.271, 2.032)	0.647
Baseline	0.727 (0.569, 0.884)	**<0.001**
Intercept	64.390 (34.608, 94.172)	**<0.001**
AIC	322.864	
AIC Model 0	324.525	
**mRT**		
Envi	-20.387 (-37.911, -2.863)	**0.024**
Baseline	0.772 (0.610, 0.934)	**<0.001**
Intercept	183.144 (85.333, 280.955)	**<0.001**
AIC	648.357	
AIC Model 0	659.758	
**SERT**		
Envi	-1.387 (-2.464, -0.310)	**0.013**
Baseline	0.660 (0.470, 0.849)	**<0.001**
Intercept	7.029 (4.135, 9.923)	**<0.001**
AIC	301.381	
AIC Model 0	308.211	


*Linear mixed models predicting executive attention (EXE), accuracy (ACC), mean reaction time (mRT), and stability of performance (SERT) on the Attention Network Task. The base model includes baseline as a covariate, subject as random factor, and environment as the fixed effect of primary interest. Follow-up stepwise modeling revealed no significant contribution of additional factors and did not improve the base models. AIC, Akaike information criteria; was used to aid model selection. AIC of Model 1 was compared with AIC of Model 0, which contained the parameters of Model 1 minus the environment factor. Bold type indicates a significant effect (p < 0.05).*

## Discussion

We investigated the restorative effect of NEs on children’s cognitive performance in a semi-randomized crossover trial. Participants exhibited faster and more stable responding while performing the ANT after 30-min walks in a NE compared to a BE, when controlling for baseline performance. We found no evidence of improved executive attention or overall accuracy. Participants rated the NE more restorative than the BE; however, ratings did not influence cognitive performance. Performance was not influenced by age, gender, or order of environment. A randomly selected sub-sample, who wore mobile eye-trackers that recorded aspects of visual processing while walking in both environments, exhibited a higher fixation rate within the NE than the BE. No differences in average fixation duration were found. While the effect size for fixations per minute was large (*d* = 1.1), interpretations remain tentative and should be met with caution given the relatively small sample size (*n* = 9). When eye-tracking outcomes were included in the linear mixed models, they did not predict cognitive performance.

Dadvand et al. (2015, 2017) showed that long-term exposure to greenspace positively influences the development of cortical regions and cognitive processes associated with performance stability in children. We support these findings by confirming that an acute exposure to a NE can also improve performance stability on the ANT in children. However, our results also showed that reaction time can also be positively influenced, which has not been previously reported.

Kaplan and Berman (2010), argued that directed attention is a cognitive resource recruited by both executive and self-regulatory processes. Self-regulatory processes, including effort allocation and the ability to maintain appropriate states of arousal, are often entwined within models of executive functioning (Diamond, 2013). Therefore, the finding that natural environments can improve the ability to control responding to meet the demands of a repetitive mundane task, such as the ANT, may not be surprising. A reduction of IIVrt may reflect an improvement in effort allocation or regulating arousal states (Wiersema et al., 2005). We recommend that future studies of restoration using multi-trial tasks report IIVrt, classic executive measures, and additional measures to explore mechanisms underlying behavioral performance, such as eye-tracking.

The large difference in fixation rates found between the environments goes against previous reports using screen-based eye-tracking. However, interpretation of this discrepancy remains ambiguous. As Berto et al. (2008), p. 186 referred to fixations as a measure of “periods of focused attention,” they concluded that fewer fixations must reflect a reduction in cognitive effort when viewing natural scenes. However, if cognitive effort refers to the ease in which attention is successfully shifted between one visual target to another, a greater number of shifts, or fixations, would reflect an environment that was more effortless to process. Fixations are traditionally known as the times at which visual information is acquired and the next target is selected (Rucci and Poletti, 2015). This would predict a greater number of fixations as a proxy for better overall apprehension of a scene. Hence, nature evokes intrinsic fascination and a desire to explore the scene while acquiring visual information, but in a way that is not taxing to the cognitive system. This may be considered intrinsic fascination at an environmental level, while intrinsic fascination at a stimulus level would predict longer durations while fixating on natural stimuli, as shown by Valtchanov and Ellard (2015). Differences in eye-tracking results may also reflect the mode of environmental exposure, where our participants experienced full sensory exposure, rather than purely visual exposures, as used in previous studies. We hope the investigation will encourage a move away from the lab and static scenes toward more immersive ethnographic research.

### Limitations

A power analysis conducted during the planning phase of the study suggested 40 participants would be required to achieve an acceptable level of power for the analysis of cognitive data. Unfortunately, due to time and logistical restraints associated with the participants’ academic schedules, we were required to cease recruitment prior to achieving this number. However, it is worth remembering that our initial power analysis was conducted using a more conservative target power of 90%. A more conventional target of 80% power would have resulted in a lower required sample size. Such an estimate may now be appropriate using the data derived from this current study involving children.

Due to the uniqueness of the sub-study involving eye-tracking, it was not possible to perform a power analysis to estimate the required sample size, even to a tentative degree. While we warn against drawing strong conclusions based on this data set alone, we encourage researchers to pursue larger studies to explore the relationships between visual processing in NEs, cognitive performance gains, and a restoration mechanism. Indeed, the eye-tracking data presented in this article may provide information necessary to conduct power estimates for larger studies in the future.

The current study would have also benefited from a more thorough analysis of the participants’ experiences during the walks to better describe the conditions necessary for restoration-related cognitive gains in children. Collado and Staats (2016) highlighted the need to explore the uniqueness of children’s experiences with their environment when considering the restoration effect in this age group. Among their considerations, they noted that children differ from adults in terms of their receptivity toward changing social contexts. One challenge of the present study was to maintain a positive social atmosphere during the walks that would promote, rather than impede, the restoration process. The peer groups in which the participants walked were identical across environments to control for social context. However, the groups were chosen at random so that participants were not necessarily placed with peers to whom they were close. To investigate the purported moderating effect that social contexts have on restorative states in children (Collado and Staats, 2016), future studies should include quantitative measures of group cohesion or relationship proximity between peers within walking groups.

Further, according to Collado and Staats (2016), a thorough audit of children’s restorative experiences should also include measures of place attachment. In the present study, most participants were familiar with both environments. However, this observation was based on informal rapport-building conversations between the participants and the experimenter, rather than being quantified in a way that could be used during analysis. It is possible to speculate that familiarity with the area in which the nature walk took place, or heightened attachment, may have enhanced the restorative value of the environment and therefore inflated the effects on cognitive performance. Whether positive place attachments and positive social contexts are necessary conditions in which children can achieve cognitive restoration during exposure to a natural environment remains unclear. However, such knowledge may have considerable bearing upon how future studies with child participants are conducted.

## Conclusion

Based on our pattern of results, which supports previous studies (e.g., Dadvand et al., 2015), we continue to speculate that the cognitive systems of children benefit in a different way to adults in response to nature exposure. Specifically, restorative environments may be more sensitive to cognitive processes related to temporal indicators of cognitive performance, (e.g., standard error of reaction time), rather than those specifically related to directed attention (e.g., executive score of the ANT). Future accounts of ART may need to be revised to accommodate this apparent difference between adults and children, should this distinction persist under future investigations. Ideally, future studies would compare children and adults within the same study. The diverging results between age groups highlight limitations of current descriptions of the restoration effect. Pairing cognitive outcome measures with additional measures that quantify the experience in NEs, such as eye-tracking, is the next step toward understanding how cognitive systems benefit from nature. Such knowledge would provide crucial information for parents and educators seeking opportunities for respite in the age of increased reliance on digital technology for child entertainment and learning.

## Ethics Statement

By law in Denmark, only biomedical studies where tissue is collected or those involving treatments with inherent risk to patients/participants must have their ethics reviewed by a Regional Ethics Board; all other research projects are exempt from applying for formal ethical approval.

## Author Contributions

All authors developed the study concept design and approved the final version for submission. Data collection was performed by MS and research assistants. Data analysis were performed by MS and RD. Interpretation of results was carried out by all authors. MS, PB, and TS drafted the manuscript.

## Conflict of Interest Statement

The authors declare that the research was conducted in the absence of any commercial or financial relationships that could be construed as a potential conflict of interest.
